# Venous Malformation Mimicking Patellofemoral Pain Syndrome in a Young Adult With Limb Length Discrepancy

**DOI:** 10.7759/cureus.115005

**Published:** 2026-08-22

**Authors:** Ron Tan, Scott Xu, Stephanie Ong

**Affiliations:** 1 Sports and Exercise Medicine, Changi General Hospital, Singapore, SGP; 2 Family Medicine, SingHealth Polyclinics, Singapore, SGP

**Keywords:** knee pain, limb length discrepancy, osteochondroma, patellofemoral pain syndrome, venous malformation

## Abstract

Limb length discrepancy (LLD) can result from a variety of underlying pathologies, and the exact etiology may be challenging to identify when patients present with common musculoskeletal complaints. We present a case of LLD in a 19-year-old gentleman likely attributed to a venous malformation at the vastus medialis, with a concomitant osteochondroma of the contralateral proximal tibia. He initially presented with intermittent right-sided knee pain. An X-ray of his bilateral lower limbs revealed an osteochondroma of the contralateral proximal tibia and a 2.5-cm LLD, with the right lower limb being shorter. He was initially diagnosed with patellofemoral pain syndrome and managed conservatively. Non-response to conservative measures prompted further imaging with magnetic resonance imaging of his right knee, which revealed a venous malformation extending from the vastus medialis muscle into the pre-femoral and Hoffa’s fat pad as well as the femoral trochlea. He subsequently underwent VenaSeal treatment of the venous malformation with improvement in pain. This case is unique for having two concomitant conditions that could potentially contribute to LLD. It also highlights the importance of having a broad differential list for patients presenting with LLD.

## Introduction

Approximately 90% of the population has a limb length discrepancy (LLD) of < 1.0 cm [1], and a majority of these patients are asymptomatic. However, greater degrees of LLD can result in an increased likelihood of symptoms and complications. Current research supports that discrepancies greater than 2.0 cm are clinically significant and require treatment [2]. Symptoms may include pain involving the back, hip, or knee, as well as gait abnormalities. Complications may include functional scoliosis and early osteoarthritis.

Furthermore, LLD can be caused by a multitude of pathologies, and it may be challenging to determine the exact etiology during the initial presentation. This is particularly relevant when patients present with musculoskeletal complaints that may resemble common pathologies, resulting in an underlying diagnosis such as a structural abnormality being overlooked.

We present this unique case of a young adult with knee pain initially suggestive of patellofemoral pain syndrome (PFPS), for which subsequent evaluation revealed a venous malformation contributing to the LLD. This case is also notable for having a concomitant osteochondroma, which can further complicate the assessment of the patient's LLD.

## Case presentation

A 19-year-old Chinese gentleman presented to the sports and exercise medicine outpatient clinic for concerns of lower LLD and right knee pain. He had been referred by the doctors in the military due to the patient’s complaint of right knee pain and examination findings of LLD. He was previously on follow-up with a podiatrist for pes planus and LLD at the age of 16 years but was non-compliant with the insoles prescribed. He had no other medical or surgical history.

In addition to the LLD, the patient also reported atraumatic, mild, right-sided anterior knee pain for the past few years. The pain was intermittent and mechanical in nature. It was exacerbated by prolonged standing, running, and squatting, and alleviated by rest. There were no symptoms of locking or instability. At the time of presentation, he was enlisted in full-time military service, which involved rigorous physical demands.

On examination, his gait was normal with no compensatory mechanisms noted such as pelvic tilting, long knee flexion, or vaulting on the short limb. His height measured 178 cm. He had bilateral flexible pes planus, calcaneovalgus, and clawing of the toes. He had a true LLD of 2 cm; his right lower limb measured 91 cm while his left lower limb measured 93 cm. Measurements were taken from the prominence of the anterior superior iliac spine (ASIS) to the lower prominence of the medial malleolus. Pain in the right anterior knee was elicited with single-leg dynamic maneuvers such as a half squat and hop. Other significant findings also included a positive Clarke’s test, which suggested a patellofemoral joint disorder, as well as a positive Galeazzi test, which suggested femoral shortening accounting for the LLD.

There was no knee effusion, and the patient had a full active range of motion of his knees. The overlying skin was normal, with no cutaneous stigmata of vascular malformations. There was no tenderness on palpation, and patellar grind was negative. Other special tests such as the anterior drawer, posterior drawer, medial and lateral collateral ligament stress tests, and McMurray’s test were also negative.

**Figure 1:**
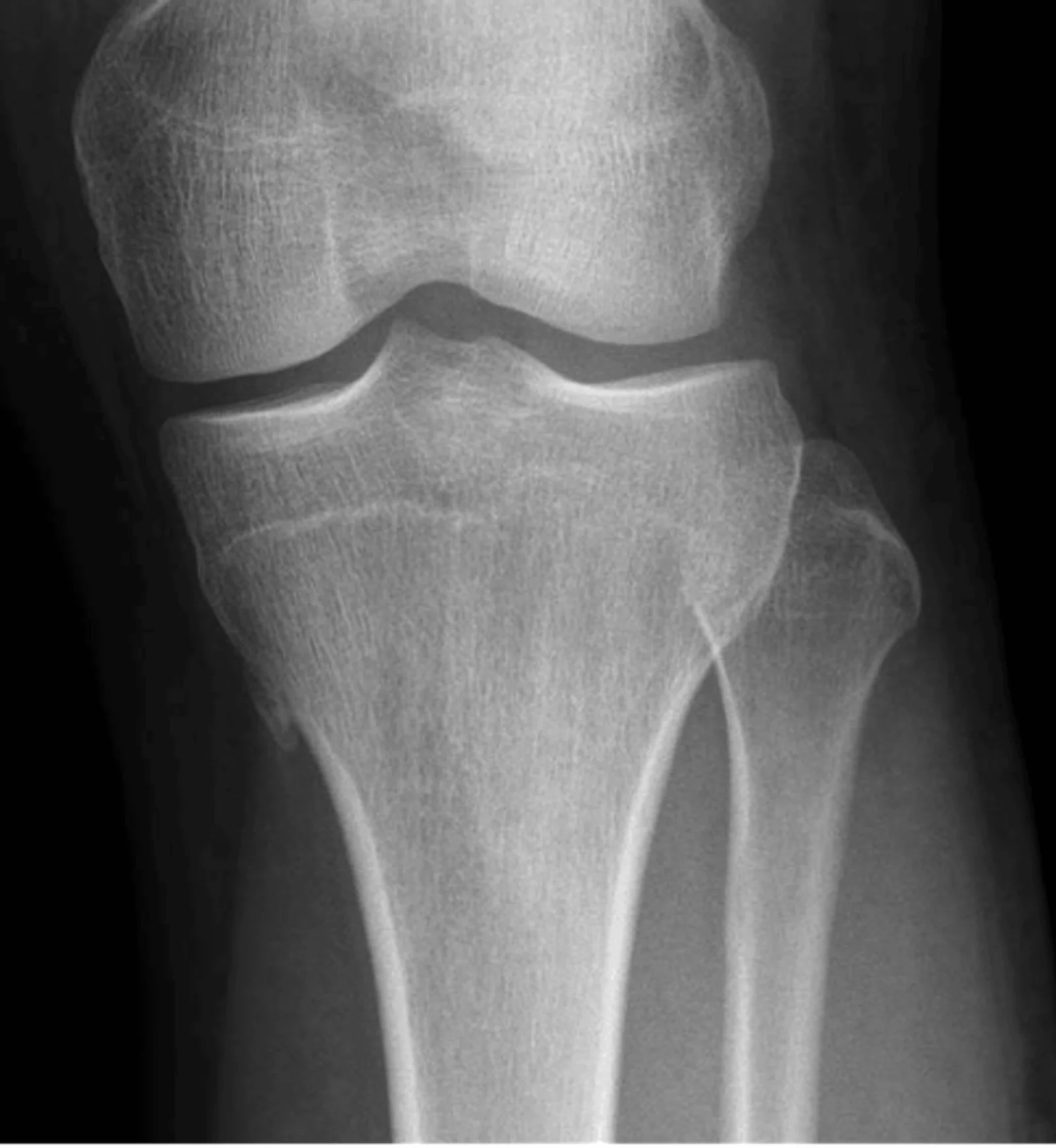
Left medial tibial bony excrescence representing an osteochondroma.

**Figure 2:**
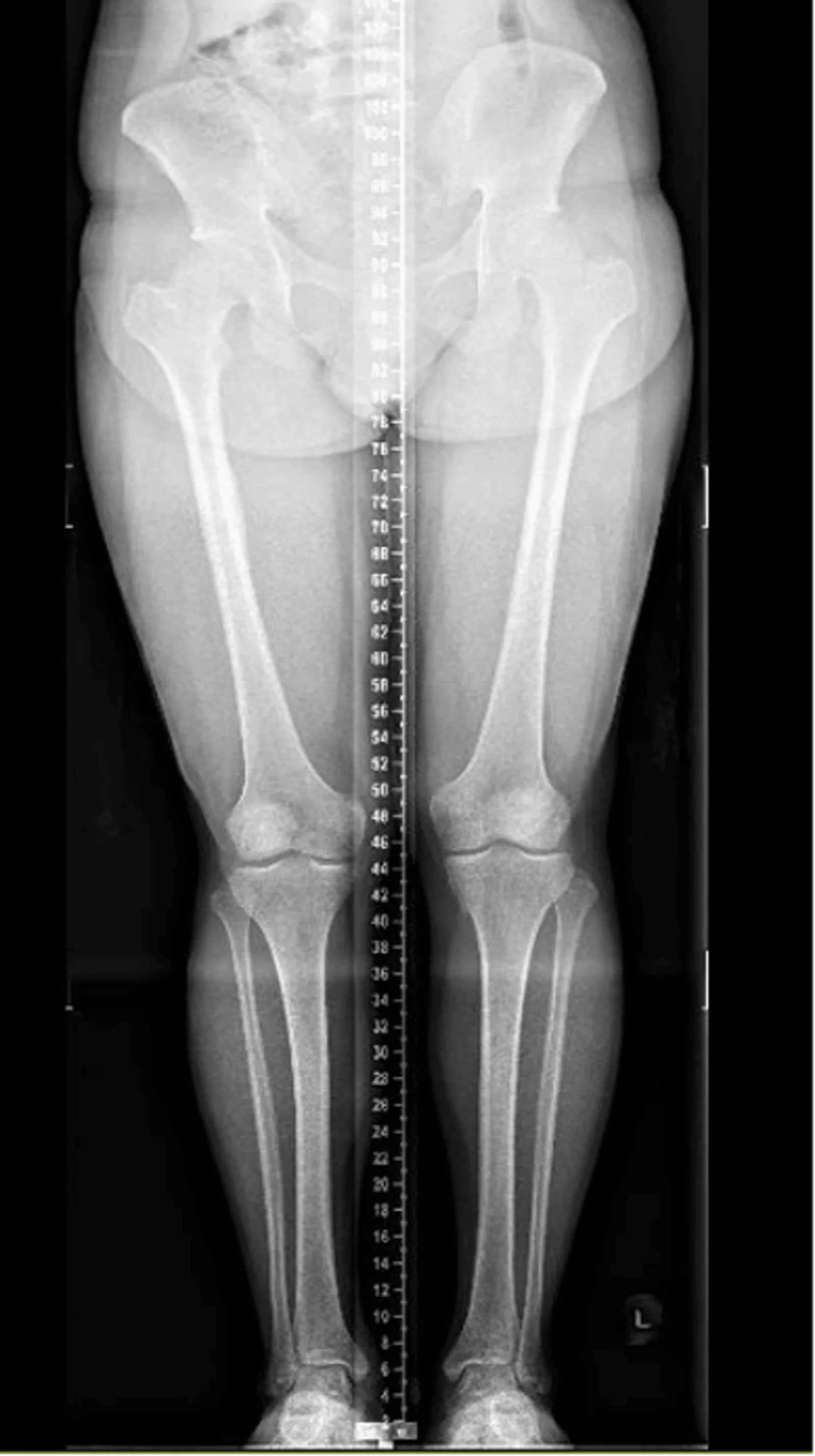
X-ray of the bilateral lower limbs showing the discrepancy in limb length.

Long leg plain radiographs of the lower limbs were performed, which noted a bony excrescence arising from the medial aspect of the proximal left tibia with corticomedullary continuation, likely an osteochondroma (Figure 1). The right lower limb measured 93.3 cm from the right ASIS to the right medial malleolus, while the left lower limb measured 95.8 cm from the left ASIS to the left medial malleolus (Figure 2). There were no other structural abnormalities noted, including over the pelvis and hips.

Differential diagnoses

Based on the patient’s current age profile, history, and examination findings, the top differential was that of right knee PFPS secondary to mechanical overload due to LLD.

Broad differentials for limb shortening, which include congenital abnormalities, trauma, infection, neurological causes, tumors or dysplasias, vascular anomalies, and inflammatory disease [3], were not apparent at this juncture, given the absence of previous trauma, joint infections, skin changes, or surgeries. Considering the positive Galeazzi test, the broad differentials to consider were those that would result in femoral shortening. This includes previous physis injuries, congenital causes such as congenital femoral deficiency, and neoplasms near the physis. The impression was that this was an incidental diagnosis of a left proximal tibia osteochondroma, as the osteochondroma was on the limb that was not shortened.

Aside from PFPS, we also considered potential structural causes in view of the knee pain with LLD, and had planned to arrange an MRI of the right knee at the follow-up visit if symptoms were not improved despite lifestyle modifications, orthotics, and physiotherapy.

Outcome and follow-up

The patient was referred to the podiatrist for insoles and heel lifts as well as the physiotherapist for lower limb strengthening and conditioning exercises. He was prescribed exercises targeted at the quadriceps, involving foam rolling and isometric exercises. He had been compliant with the physiotherapy sessions and reported to have performed his home exercises as well. On follow-up six months later, the patient continued to have persistent right-sided knee pain, and he was subsequently scheduled for an MRI of the right knee.

**Figure 3:**
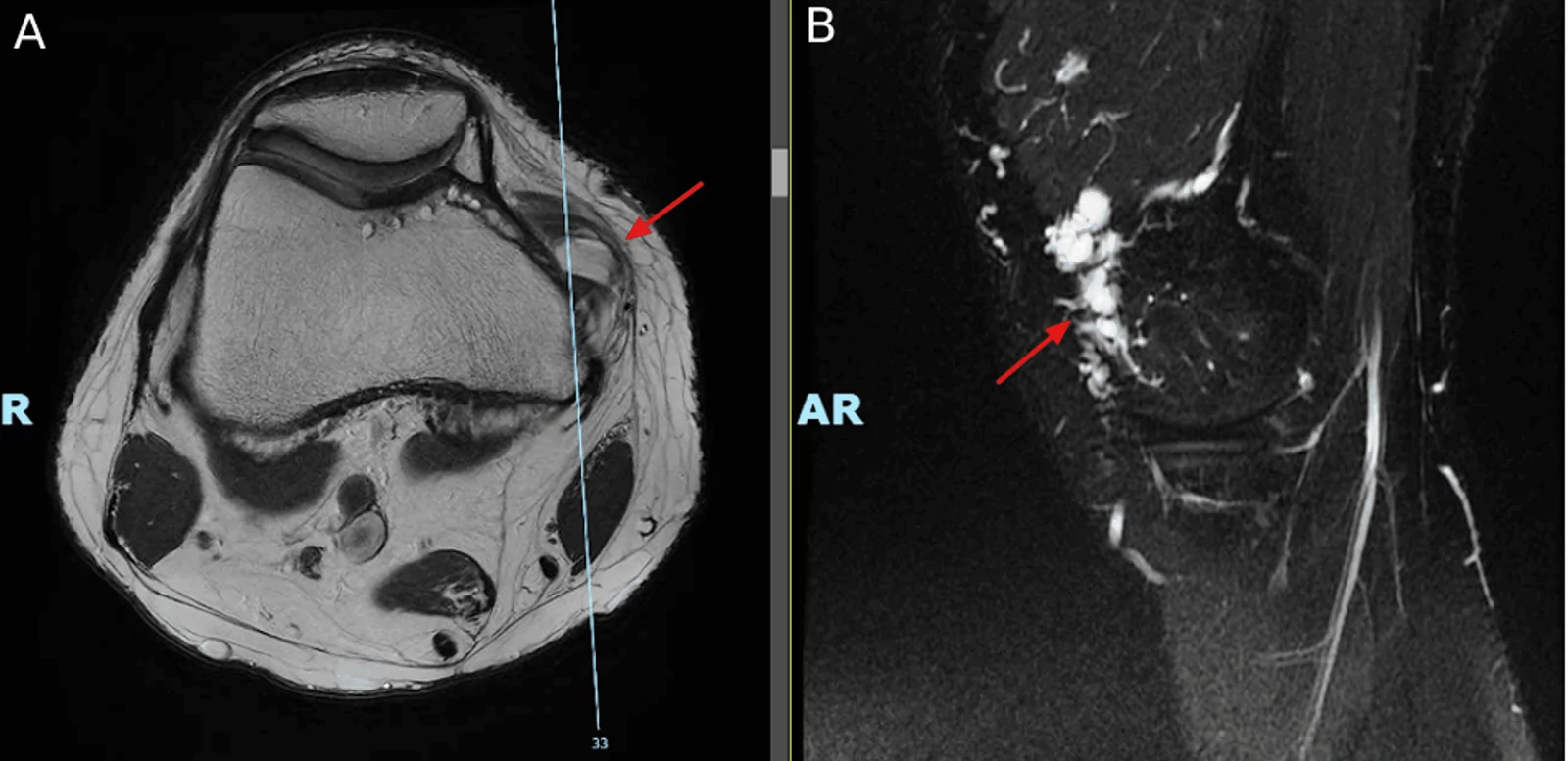
MRI of the right knee showing clusters of dilated, tortuous vessels, some focally dilated, extending from the vastus medialis muscle into the pre-femoral and Hoffa's fat pad, as well as extending into the femoral trochlea, suggestive of a slow-flow vascular malformation such as a venous malformation. (A) Transverse plane. (B) Sagittal plane.

**Figure 4:**
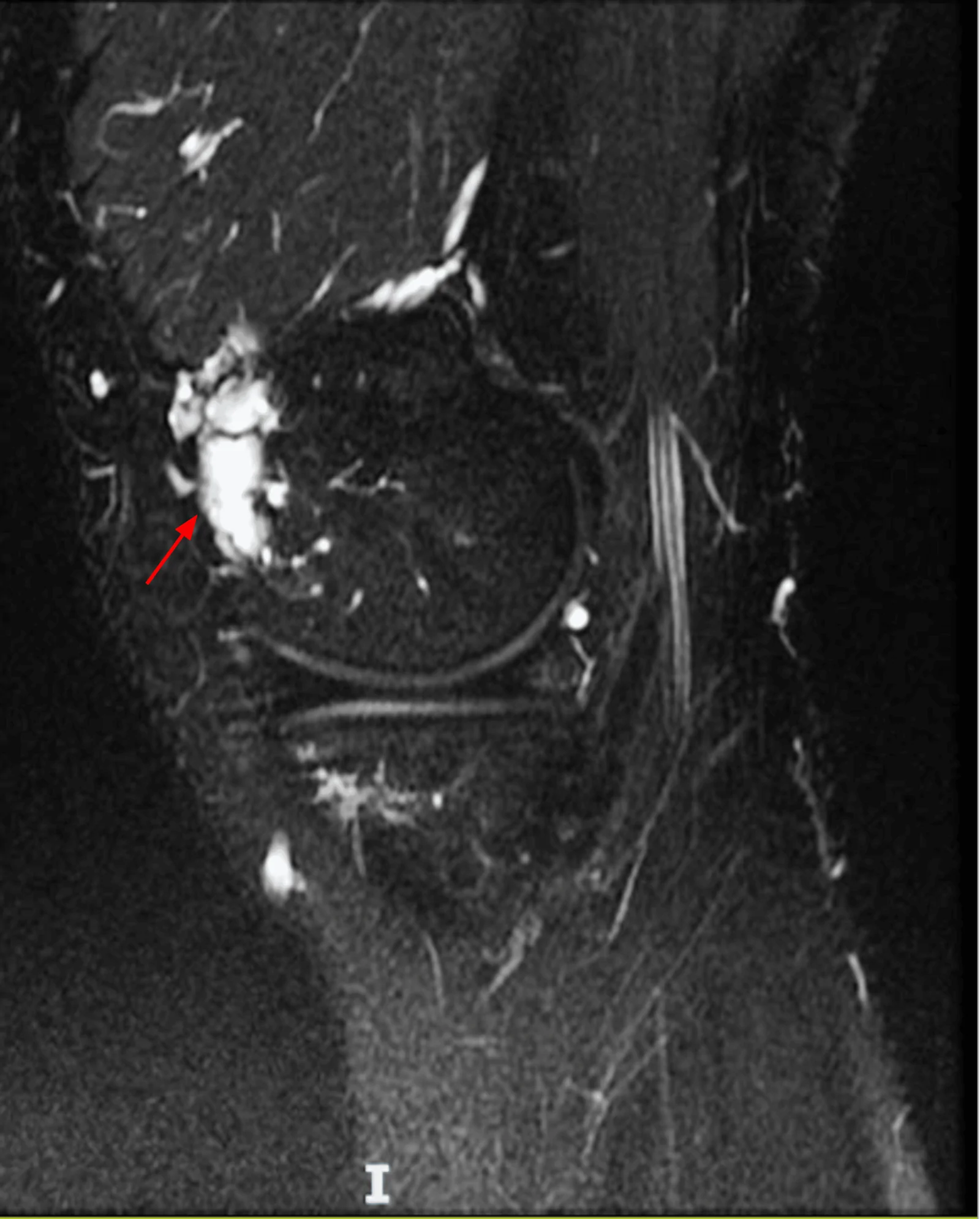
MRI of the right knee demonstrating a slow-flow vascular malformation.

**Figure 5:**
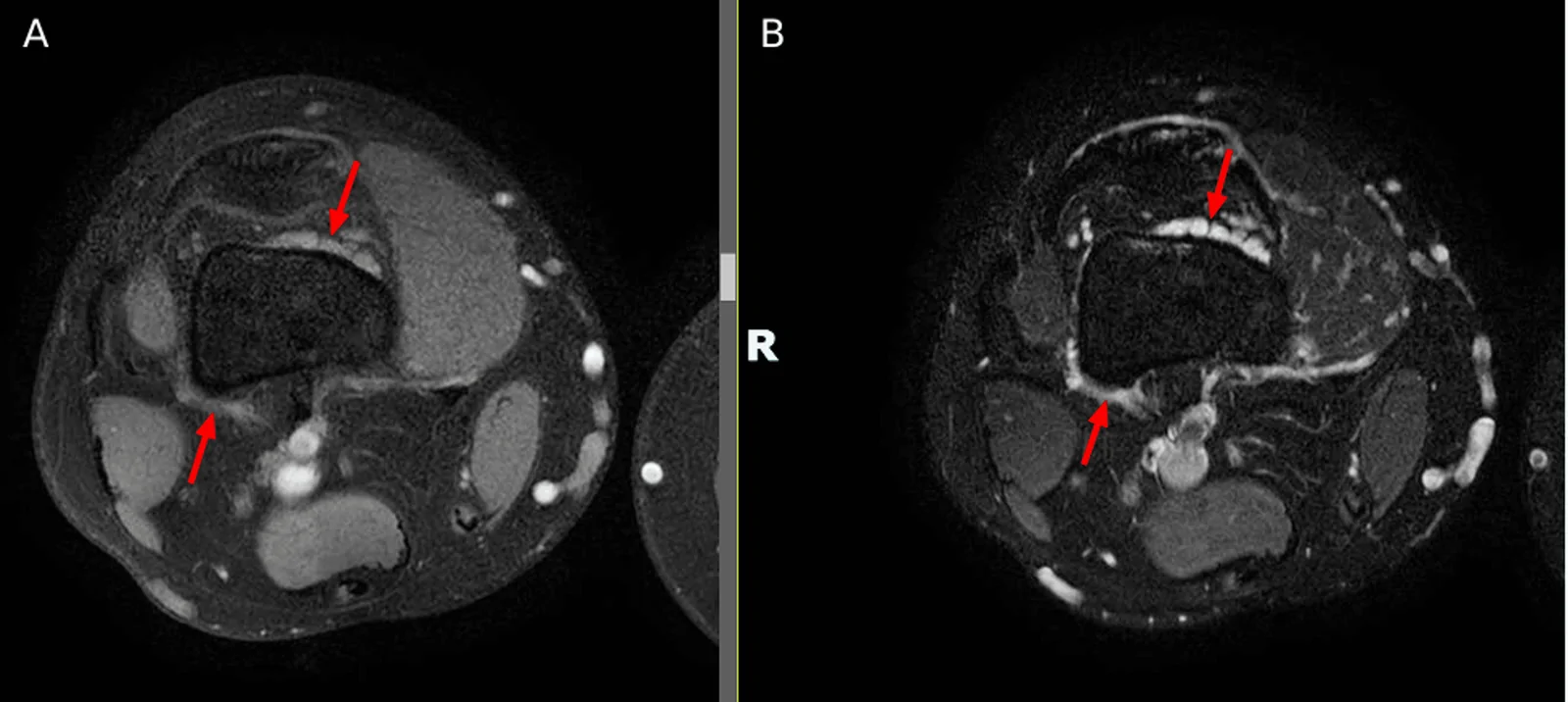
MRI of the right knee demonstrating features of a venous malformation. (A) Intermediate signal intensity on T1-weighted imaging. (B) Hyperintense signal on T2-weighted imaging, consistent with the characteristics of venous malformations.

**Figure 6:**
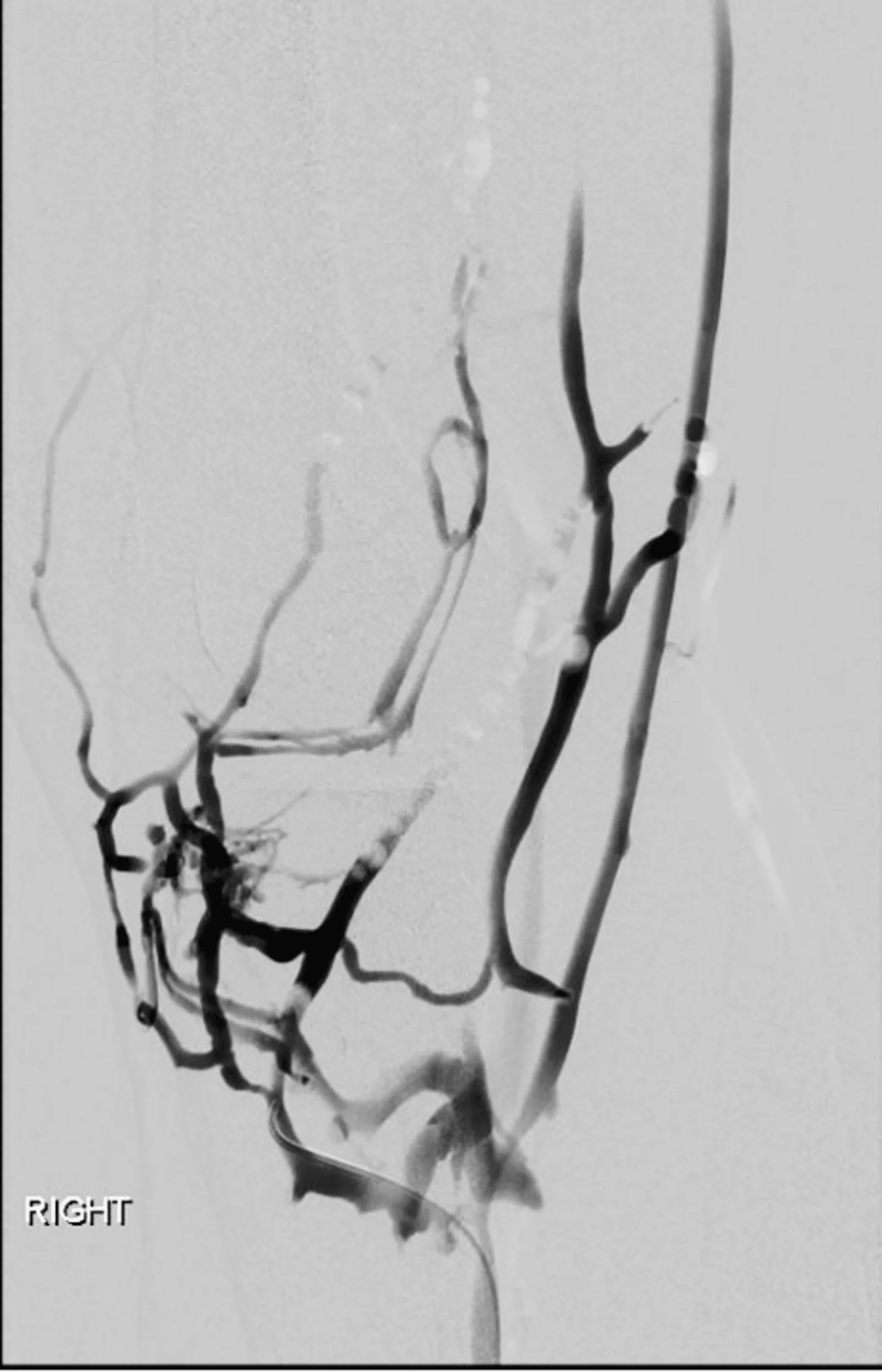
Intraoperative angiogram demonstrating a small tangle of beaded-appearing veins, likely corresponding to the intra-articular venous malformation identified on prior MRI.

MRI of the right knee noted clusters of dilated tortuous vessels, some focally dilated, extending from the vastus medialis muscle into the pre-femoral and Hoffa’s fat pad, and the femoral trochlea (Figures 3, 4). These lesions demonstrated intermediate signal intensity on T1-weighted imaging and hyperintense signal on T2-weighted imaging, consistent with the typical imaging characteristics of a venous malformation (Figure 5). These findings suggested the presence of a slow-flow vascular malformation, potentially a venous malformation. In view of persistent right-sided knee pain that was likely attributed to the venous malformation, the patient was referred to the vascular surgeon for surgical intervention and underwent a right lower limb diagnostic angiogram. The angiogram noted several deep venous malformations, which might have been intra-articular, and were supplied by the long saphenous vein (Figure 6). A shared decision was made between the patient and the surgeon to proceed with a long saphenous vein VenaSeal. The surgery was uneventful, and the patient was discharged well on postoperative day 1. He was reviewed one month after surgery by both the vascular surgeons and sports medicine physicians and reported improvement in his right knee pain. He was given an open-date appointment by both specialties. The patient was keen to consider limb-lengthening corrective procedures, and he was referred to the orthopedic surgeons for further discussion.

## Discussion

The etiologies of LLD are varied and can be broadly classified into congenital and acquired causes. Congenital causes include conditions such as developmental dysplasia of the hip, fibular hemimelia, bowing of the tibia, and clubfoot. Acquired causes include trauma to the growth plate, infection, avascular necrosis, functional scoliosis, polio, vascular malformations, as well as benign and malignant tumors [3]. Clinical measurement of the LLD reported a 2-cm discrepancy, whereas radiographic imaging revealed a 2.5-cm discrepancy. This could be due to factors such as patient positioning, pelvic tilt, and clinician technique. The radiographic assessment of 2.5 cm as the LLD is adopted for further discussion, as it is a more valid and reliable method of assessing LLD [4]. The patient had an LLD of 2.5 cm, which is significant and is generally perceivable by almost all patients [5]. Given that he had only experienced lower limb symptoms in late childhood, an acquired cause would be more likely than a congenital cause. A patient’s height can also affect how they perceive the degree and impact of the LLD, with the same discrepancy affecting those of a shorter stature to a greater degree [3]. The patient’s height was above the average height for his demographic [6], potentially explaining why he was less affected by the LLD. Aside, his degree of LLD was 2.5 cm, which was the borderline consensus upper limit of what is considered significant based on existing literature. Prior to the knowledge of the venous malformation, there was no clear cause to explain the LLD. Hence, the patient was treated for his LLD with non-pharmacological measures such as a shoe lift. Treatment of LLD generally involves a shoe lift once the discrepancy approaches 2 cm [7]. Shoe lifts generally function well up to approximately 3 cm, beyond which the shoe may become heavy and awkward [3]. In more severe cases of LLD where the patient’s function is affected, such as with gait abnormalities, surgical interventions can be considered to achieve selective growth plate closure in patients prior to skeletal maturity, or bone shortening for patients who have achieved skeletal maturity [7].

The patient was also diagnosed with an osteochondroma of the left proximal tibia, which may have contributed to his LLD. An osteochondroma is a cartilage-capped bony spur arising on the external surface of a bone. They mostly occur spontaneously and account for approximately 30% of benign bone tumors. Patients may complain of pain, decreased range of motion, and a notable lump. Osteochondromas can potentially affect the adjacent growth plate, resulting in valgus or varus deformities of the nearby joint or shorter stature of the affected limb [8]. However, a recent study found that solitary osteochondromas involving the proximal tibia did not cause clinically significant deformity of the lower extremity [9]. This case further supports this finding, as the osteochondroma was located at the proximal tibia and involved the left lower limb, which was not shortened in comparison to the right lower limb. Hence, it is more likely that the osteochondroma was an incidental finding, and less likely to have accounted for the patient's LLD. On reflection, the venous malformation was the most likely contributor to the patient's LLD. This is supported by the patient's clinically significant LLD, the presence of a venous malformation in the affected lower limb, the association between venous malformation and LLD, and the absence of other potential structural causes for the LLD.

Vascular malformations can be classified into fast-flow and slow-flow anomalies. Fast-flow vascular malformations consist of arterial malformations, arteriovenous fistulae, or arteriovenous malformations. Slow-flow vascular malformations include venous, lymphatic, and capillary malformations [10]. MRI findings suggestive of a venous malformation are intermediate T1, bright T2, and absent flow voids, with normal feeding arteries [11]. These were seen on the MRI of this patient. Venous malformations are the most common anomalies of the lower limb, and characteristics include phleboliths, morning pain, and emptying with compression [12]. The overlying skin may demonstrate color changes depending on the depth of the vascular malformation. This case presented a diagnostic dilemma as there were many features that pointed toward PFPS rather than vascular malformation, such as the patient’s age, current occupation, and positive Clarke’s test on examination. Lower limb examination also did not note any specific findings that may allude to a vascular malformation, such as localized tenderness, swelling, or overlying skin changes. The lack of overlying skin changes in this patient may be attributed to the depth of the venous malformation, which was within the vastus medialis. Cutaneous findings of vascular anomalies can also be challenging to differentiate in patients with darker Fitzpatrick skin types [13] and potentially also account for the lack of cutaneous findings in this patient.

In addition, the patient’s knee pain was more likely due to local pressure and invasion by the vascular malformation, with a lesser contribution by PFPS. This is due to the lack of improvement in the patient’s symptoms despite a six-month duration of physiotherapy and activity modification, addressing the problem of PFPS. Physiotherapy has been demonstrated to result in strong pain reduction and improvements in activity levels for patients with PFPS, with significant short-term effects seen at the 12-week interval [14]. In addition, physiotherapy involving quadriceps-strengthening exercises has been shown to improve pain levels both immediately post treatment and at a longer follow-up duration at the 12-month interval [15]. The patient received appropriate physiotherapy exercises focused on quadriceps isometric strengthening, for an adequate duration of six months. However, this did not result in improvement in pain outcomes. On the other hand, the surgical intervention for the venous malformation resulted in rapid pain relief within one month. This supports the diagnosis of the vascular malformation as the main cause of the patient’s symptoms.

Separately, case reports of vascular malformations and their impact on LLD have been published in the younger pediatric population and may be associated with rare congenital conditions such as Klippel-Trenaunay syndrome and Servelle-Martorell syndrome. Klippel-Trenaunay syndrome results in limb lengthening, whereas Servelle-Martorell syndrome results in limb shortening [10]. Klippel-Trenaunay syndrome is characterized by the triad of capillary malformations with port-wine stains, venous malformations, and limb hypertrophy [16]. In Klippel-Trenaunay syndrome, the port-wine stain is usually evident at birth, and the full syndrome develops during the period of growth [9]. While this patient had venous malformations and LLD, there were no port-wine stains or limb hypertrophy noted clinically, making this diagnosis less likely. Servelle-Martorell syndrome is characterized by venous or arterial malformations associated with bone hypoplasia and soft tissue hypertrophy [17]. The patient had neither of these. A review of existing literature reports that the average age of diagnosis of vascular malformations is in the pediatric age group [18,19]. This is similar to the mean age of diagnosis of solitary osteochondromas [5]. These are less commonly reported in an older age group. In addition, a recent literature review noted that there were no previous case reports on concomitant osteochondroma and vascular malformation in the lower limbs, manifesting as LLD. This case highlights the importance of longitudinal follow-up while keeping a broad list of differentials and considering advanced imaging in the event of persistent unexplained symptoms.

## Conclusions

This case illustrates a rare presentation of LLD in a young adult due to a venous malformation, with an incidental contralateral osteochondroma. It underscores the importance of maintaining a broad differential list when evaluating LLD, particularly in patients with persistent symptoms unresponsive to conservative management. The case also highlights the diagnostic challenges of deep vascular malformations in the absence of cutaneous findings. Clinicians should consider advanced imaging in patients with persistent or unexplained symptoms despite appropriate initial assessment and management.
